# Mechanism of Reduced Glutathione Induced Lysozyme Defolding and Molecular Self-Assembly

**DOI:** 10.3390/foods12101931

**Published:** 2023-05-09

**Authors:** Dashan Guo, Yuwei Hou, Hongshan Liang, Lingyu Han, Bin Li, Bin Zhou

**Affiliations:** 1 Cooperative Innovation Center of Industrial Fermentation (Ministry of Education & Hubei Province), National “111” Center for Cellular Regulation and Molecular Pharmaceutics, School of Biological Engineering and Food, Hubei University of Technology, Wuhan 430068, China; dashan_66@163.com (D.G.); v82798972900@163.com (Y.H.); 2 College of Food Science and Technology, Huazhong Agricultural University, Wuhan 430070, China; lianghongshan@mail.hzau.edu.cn (H.L.); libinfood@mail.hzau.edu.cn (B.L.); 3 Key Lab of Biotechnology and Bioresources Utilization of Ministry of Education, College of Life Science, Dalian Minzu University, Dalian 116600, China; hly@dlnu.edu.com

**Keywords:** reduced glutathione, lysozyme, defolding, self-assembly

## Abstract

The distinctive assembly behaviors of lysozyme (Lys) feature prominently in food, materials, biomedicine, and other fields and have intrigued many scholars. Although our previous work suggested that reduced glutathione (GSH) could induce lysozyme to form interfacial films at the air/water interface, the underlying mechanism is still obscure. In the present study, the effects of GSH on the disulfide bond and protein conformation of lysozyme were investigated by fluorescence spectroscopy, circular dichroism spectroscopy, and infrared spectroscopy. The findings demonstrated that GSH was able to break the disulfide bond in lysozyme molecules through the sulfhydryl/disulfide bond exchange reaction, thereby unraveling the lysozyme. The β-sheet structure of lysozyme expanded significantly, while the contents of α-helix and β-turn decreased. Furthermore, the interfacial tension and morphology analysis supported that the unfolded lysozyme tended to arrange macroscopic interfacial films at the air/water interface. It was found that pH and GSH concentrations had an impact on the aforementioned processes, with higher pH or GSH levels having a positive effect. This paper on the exploration of the mechanism of GSH-induced lysozyme interface assembly and the development of lysozyme-based green coatings has better instructive significance.

## 1. Introduction

Lysozyme (Lys) is a natural antibacterial protein broadly occurring in living organisms (egg white, saliva, tears, etc.) and is utilized in a wide variety of fields, including food, cosmetics, medicine, biological materials, and other domains [1]. With the maturation of high-efficiency lysozyme co-production extraction technology, especially the industrialization of cutting-edge technology such as synthetic biology, the production cost of lysozyme will be greatly reduced. It is commonly reported that Lys engages in unique self-assembly behaviors in response to distinctive environmental conditions. For example, Lys will undergo fibrosis reaction after a period of acid heat treatment [2,3,4]. In addition, tris(2-carboxyethyl) phosphine hydrochloride (TCEP) or dithiothreitol as a strong reducing agent is prone to break the disulfide bonds of the molecules of globulin (β-lactoglobulin, insulin, Lys, bovine serum albumin, etc.) and further alter the conformation of the protein. This will prompt the protein to go through a rapid phase transition in the mild water phase environment and eventually self-assemble into a dense transparent film with an amyloid structure [5,6]. Moreover, it is feasible to carry out further functional modifications on the basis of abundant active groups of the protein. These modifications are involved in the construction of superhydrophobic surfaces [7], antibacterial coatings [8], filtration membrane modifications [9], delivery system fabrication [10], etc., and can be extensively employed in biomedicine, sensing, and other industries. However, due to safety problems that severely constrain their utilization in the food industry, TCEP and dithiothreitol do not conform to the concept of green and sustainable industrial development. Coincidentally, we found out that food additives with a variety of biological activities—e.g., reduced glutathione (GSH) mixed with Lys—also induce an intriguing assembly behavior of Lys, i.e., the formation of an interface membrane visible to the naked eye at the air/water interface [11]. It can be easily transferred to different base surfaces for functional modification. Nevertheless, it remains to be determined how GSH affects the molecular conformation of Lys and how its changes act on the interfacial assembly behaviors. That understanding is also obscured by external factors such as the pH and the concentration of the reactants that affected this fascinating process. Figuring out the internal mechanism is of great guiding significance for regulating the assembly process and the properties of the interfacial film, which is advantageous to promote its applications in food or other fields.

Hence, this work applied a combination of spectroscopy, interfacial rheology, and microscopic imaging methods to analyze the influence of key factors (pH, concentration) on the process of Lys folding and conformational transformation induced by GSH, as well as the interfacial tension and microscopic process of Lys assembly. This research is aimed at fostering theories of regulation and methods of protein assembly and furnishing industrial applications of protein interface assembly with theoretical guidance that can also serve as updated principles and approaches for the innovation of protein functional coatings.

## 2. Materials and Methods

### 2.1. Materials

Lysozyme (hen egg white, ~20,000 U/mg) and GSH were purchased from Yuanye Bio-Technology Co., Ltd. (Shanghai, China). N-(1-pyrenyl) maleimide (NPM) and 8-anilino-1-naphtalenesulfonic (ANS) were obtained from Aladdin Biochemical Technology Co., Ltd. (Shanghai, China). 4-(2-hydroxyethyl)-1-piperazineethanesulfonic acid (HEPES) buffer and TCEP was purchased from Solarbio Science and Technology Co., Ltd. (Beijing, China). All reagents and solvents were of analytical grade, and ultra pure water (18.3 MΩ·cm) was used in the entire experiment.

### 2.2. Preparation of Partially Unfolded Lys Nanofilm

First, Lys-GSH mixed solutions were prepared. With HEPES buffer solution as the solvent, Lys solution (7 mg/mL) and diverse concentrations of GSH solution (1.75, 3.5, 7, 14 mg/mL) were prepared, respectively. Different groups of Lys-GSH mixed solution were produced according to the volume ratio of 1:1; GSH solution (7 mg/mL) was adjusted to the desired pH value (pH = 7, 8, 9, 10) using NaOH, and then was mixed with Lys solution evenly under the same volume and concentration to obtain the other groups of Lys-GSH solution.

After mixing the HEPES buffer solution of Lys (7 mg/mL) with the HEPES buffer solution of GSH (7 mg/mL, pH = 9) in equal volume, the mixture solution of Lys-GSH was dropped on a glass plate treated with piranha solution using a pipet-gun, on which a colorless and transparent film was generated at 25 °C for 2 h. Finally, when immersed in water, a layer of protein film could be observed floating on the water surface, and then was picked up with the covered glass plate. These films were dried at room temperature after utilizing filter paper to absorb the excess water, yielding Lys/GSH films.

### 2.3. NPM Fluorescence Analysis

Briefly, 200 μL of N-(1-pyrenyl) maleimide (NPM) solution (15 mM, in DMF) was mixed with 1400 μL of lysozyme (7 mg/mL) HEPES buffer, and then the cuvet was put into the fluorescence spectrometer. Subsequently, 1400 μL of GSH solution (7 mg/mL, pH = 9) was added to keep track of the change in fluorescence intensity over time. The same concentration of natural Lys solution was used as the control. The instrument parameters were set as follows: 330 nm was the excitation wavelength; the emission wavelength was 380 nm, and the slit widths were 5 nm.

### 2.4. Trp Fluorescence Analysis

Equal volumes of Lys solution (7 mg/mL) and GSH solution (7 mg/mL, pH = 7, 8, 9, 10) were mixed to obtain 2 mL of partially unfolded Lys solution. The fluorescence emission spectrum of tryptophan (Trp) in Lys molecules was detected with a fluorescence spectrophotometer (F-4700, Hitachi Limited, Hitachi, Japan). Instrument parameter settings: excitation wavelength and emission wavelength were 285 nm and 340 nm, respectively; the bandwidths of excitation and emission slits were set as 5 nm. Meanwhile, the same concentrations of natural Lys solution and GSH solution were established as controls.

### 2.5. ANS Fluorescence Probe Analysis

ANS was used to analyze the exposure differences of hydrophobic groups of Lys at various pH and concentrations. Briefly, 1400 μL of Lys buffer solution (7 mg/mL) was added to 200 μL ANS solution containing 300 μM solute, successively adding 1400 μL GSH solution to the full mixture after 10 min. The emission wavelength at 475 nm of the samples was determined with a fluorescence spectrophotometer (F-4700, Hitachi Limited, Hitachi, Japan) with excitation spectra at 350 nm. The bandwidths of excitation and emission slits were set at 5 nm. Natural Lys solution and GSH solution served as the control groups at the same concentration.

### 2.6. Circular Dichroism (CD) Spectrum

The secondary structure of proteins can be qualitatively described by far UV-circular dichroism (J-1500, Jasco, Tokyo, Japan). α-Helix presented two characteristic negative peaks at 222 nm and 208 nm in the CD band, and β-sheet showed negative peaks at 215 nm. A 200 μL amount of Lys solution partially unfolded through dialysis was injected into a 1 mm optical path colorimetric dish, and then CD curves were measured. Instrument parameter settings: the scanning wavelength range was from 190 to 240 nm, and the bandwidth was 2 nm. Lys/GSH nanofilms were transferred to a hollow substrate and were then investigated by far UV-circular dichroism with a scanning wavelength range of 190–240 nm at a 2 nm bandwidth. 

### 2.7. Attenuated Total Reflection Fourier Transform Infrared (ATR-FTIR)

The Lys/GSH film was transferred to the quartz sheet and tested with an ATR-FTIR spectrometer (Nicoletetis-50, Thermo Fisher Scientific, Waltham, MA, USA) with 128 scanning times. The infrared curves of the amide Ⅰ and Ⅱ bands of the samples were fitted to calculate the secondary structure content of Lys/GSH films. PeakFit (peakfit v4 version, seasolve software Inc., San Jose, CA, USA) and OMNIC (version 8.2, Thermo Fisher Scientific Inc., Waltham, MA, USA) were used in the process with spectra acquired from each sample [12].

### 2.8. Turbidity Measurement

The turbidity of the unfolded Lys mixture was measured using a UV spectrophotometer (UV-2600, Shimadzu, Suzhou, China) with a wavelength setting of 590 nm using a 1 cm light range cuvette. Turbidity is calculated by the transmittance (T) according to the following equation:

(1) $$T=-\ln\left(\frac{I}{I_{0}}\right)$$

where I and I_0_ are the transmitted light intensity and incident light intensity, respectively.

### 2.9. Zeta-Potential and Particle Size Analysis

The surface charge distribution of colloidal particles in Lys/GSH mixed solution was measured with a potential particle size meter (Zetasizer Nano-ZS, Malvern, UK). Lys solution was mixed with reduced GSH solution in equal volume and measured after the reaction for 30 min.

Dynamic light scattering (DLS) measurements were used to track the distribution of particle sizes. Equal amounts of Lys solution and reduced GSH solution of 0.5 mL were mixed evenly and measured immediately. The measurements were taken every 30 min and stopped at 60 min. All of the above operations were performed at 25 °C.

### 2.10. Determination of Surface Tension

A surface tensimeter (DCAT 25, DataPhysics Instruments GmbH, Filderstadt, Germany) was applied to place the ring into 25 mL sample solution at 25 °C, submerging the ring to 3 mm at a test rate of 1 mm/s. The ring method was applied to measure the surface tension changes of Lys/GSH mixed solution prepared under different concentrations and pH conditions. Each group was measured three times.

### 2.11. Atomic Force Microscopy (AFM)

The films formulated under different concentrations, pH values, and incubation times were transferred to clean silicon wafers and afterward dried naturally. AFM (Dimension Icon, Bruker, Billerica, MA, USA) was utilized to investigate the thickness and roughness of Lys/GSH films. Scanning parameters: scanning mode: tap mode, scanning frequency: 1 μm/s.

### 2.12. Statistical Analysis

All experiments were performed three times, and the results were presented as mean ± standard deviation. The data analysis was conducted using SPSS (20.0) software (IBM, Chicago, IL, USA). The statistical significance was examined by applying the analysis of variance (ANOVA).

## 3. Results

### 3.1. Reduction of Disulfide Bonds in Defolded Lys

**Figure 1:**
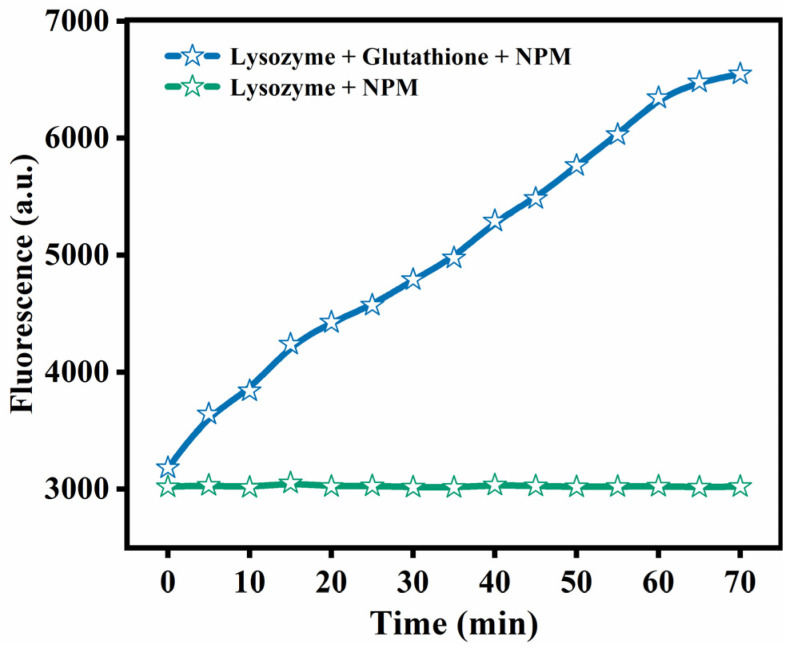
NPM assay for native Lys and partially unfolded Lys.

NPM is a fluorescent reagent that is frequently utilized in the detection of derivatives of compounds containing thioamides [13]. The curve of NPM fluorescence intensity over time is presented in Figure 1. The fluorescence was significantly enhanced in the presence of GSH and reached its peak at nearly 70 min, while the fluorescence intensity of natural Lys remained unchanged. This could also be because GSH may have broken the disulfide bond between Lys and released free sulfhydryl groups, which was the cause of a significant increase in the fluorescence strength at 380 nm [14].

**Figure 2:**
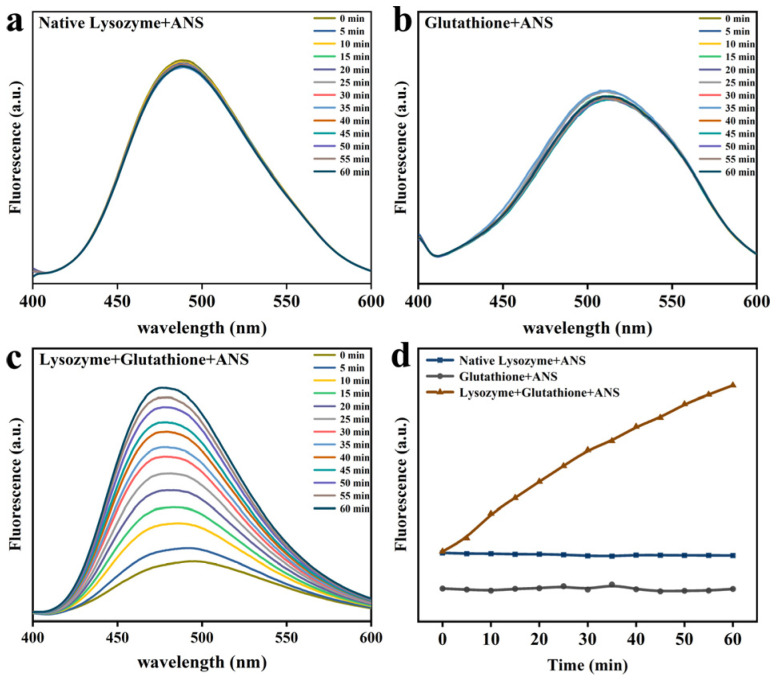
The ANS fluorescence assay for native Lys (**a**), GSH (**b**), Lys and GSH (**c**) at different incubation times. (**d**) The change of ANS fluorescence intensity for native Lys, GSH, and partially unfolded Lys as a function time of the reaction time.

**Figure 3:**
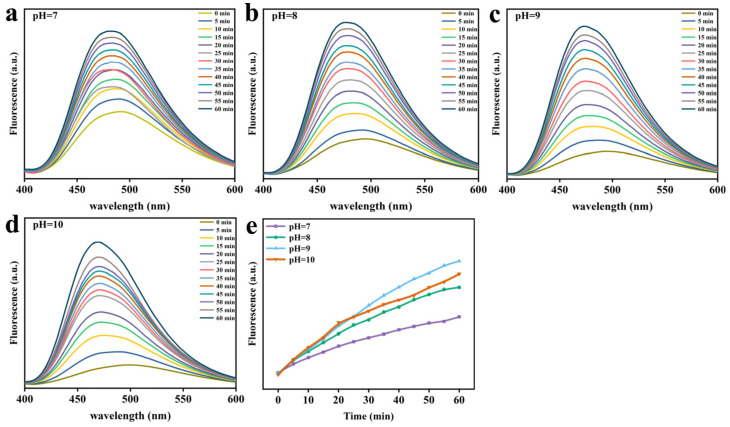
ANS fluorescence intensity characteristic of Lys upon mixing the Lys buffer with GSH buffer at different pH values. (**a**) pH 7. (**b**) pH 8. (**c**) pH 9. (**d**) pH 10. (**e**) The change in ANS fluorescence intensity at 475 nm as a function of the reaction time at four different pH values.

**Figure 4:**
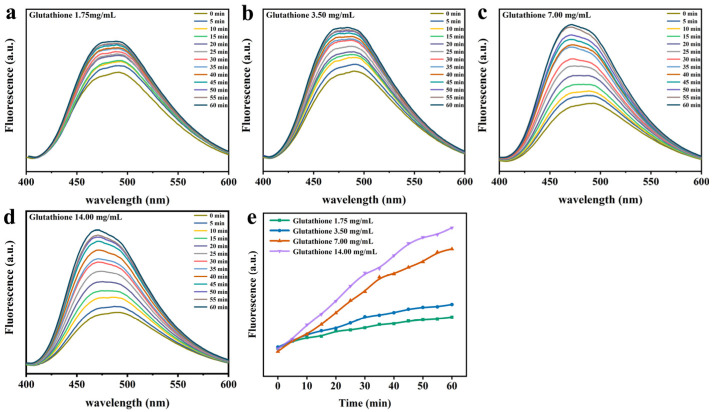
ANS fluorescence intensity characteristic of Lys upon mixing the Lys buffer with GSH buffer at different concentrations. (**a**) 1.75 mg/mL. (**b**) 3.5 mg/mL. (**c**) 7 mg/mL. (**d**) 14 mg/mL. (**e**) The change in the ANS fluorescence intensity at 475 nm as a function of the reaction time at four different concentrations of the GSH buffer.

ANS is a probe that monitors protein folding, interacting primarily with hydrophobic sites exposed to solvents. As evident from Figure 2, the fluorescence intensity of ANS was enhanced over time in the presence of GSH in comparison to that of natural Lys. This is because a considerable quantity of Lys was deconvolved and exposed to hydrophobic groups with the prolongation of time, engaging in growing numbers of action sites that bind specifically to ANS [11]. As depicted in Figure 3, it was found that the fluorescence intensity was stronger at the higher pH values, and Lys underwent unfolding and exposure to hydrophobic groups more rapidly. On top of that, fluorescence intensity was close to the isoelectric points of Lys at pH 10, the degree of ionization of the partial Lys molecular chain was undermined, and the aggregation and assembly of the folding molecules accelerated greatly. Hence, fluorescence intensity suffered a gradual reduction as a result of sedimentation initiated by excessive aggregation. Similarly, the fluorescence intensity also showed an upward trend, progressively varying with the rise in GSH concentration (Figure 4), and blue shift occurred at the maximum absorption peak λ_max_. This could be ascribed to the interruption of disulfide bonds by GSH, which led to the partial defolding of Lys and the increasing hydrophobic force between Lys molecules due to the exposure to numerous hydrophobic groups with the extension of time.

**Figure 5:**
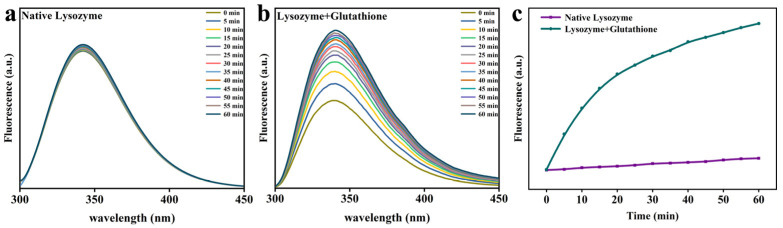
Intrinsic tryptophan fluorescence assay for native Lys (**a**), Lys and GSH (**b**) at different incubation times. (**c**) The change of Intrinsic tryptophan fluorescence intensity for native Lys and partially unfolded Lys as a function of the reaction time.

**Figure 6:**
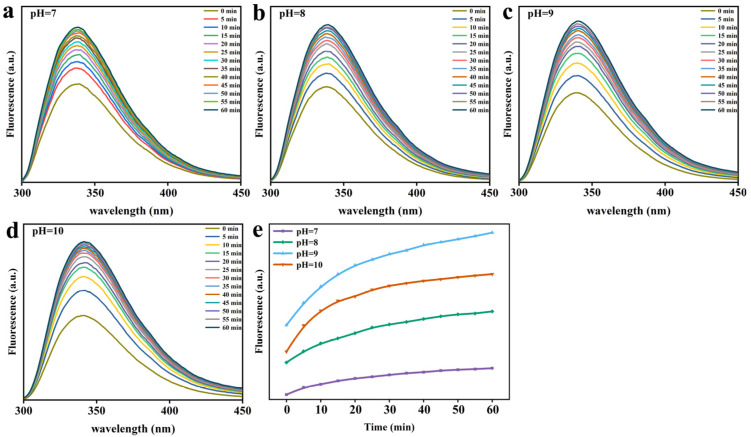
The Trp fluorescence intensity characteristic of Lys upon mixing the Lys buffer with GSH buffer at different pH values. (**a**) pH 7. (**b**) pH 8. (**c**) pH 9. (**d**) pH 10. (**e**) The change in Trp fluorescence intensity at 340 nm as a function of the reaction time at four different pH values.

Figure 5 and Figure 6 demonstrate that the time-resolved fluorescence spectra of Trp were mutually verified with the above experimental results. As reported in the literature, the natural conformation of Lys molecules consisted mainly of six Tryptophan (Trp) residues containing hydrophobic microregions. Owing to the damage to the tertiary structure of Tryptophan, the hydrophobic groups such as Trp would be exposed to the polar environment and provide the primary driving force for the gathering process [15,16]. As can be seen from Figure 5, the fluorescence of Trp in the bulk phase after the addition of reduced GSH expanded at 380 nm as against natural Lys. As the pH value rose (Figure 6), the maximum emission wavelength (λ_max_) also experienced a red shift, since the fluorescence intensity ascended. This stems from the fact that the higher pH value promotes the exposure of hydrophobic groups as well as the transfer of Trp in Lys molecules to the polar environment. In addition, the self-assembly of Lys molecules is too fast at pH 10, contributing to sedimentation caused by over-aggregation and the reduction of fluorescence intensity of Trp. The fluorescence in the remaining mixed solution increased with the pH, and a higher pH made hydrophobic groups more visible, which in turn rendered hydrophobic aggregation stronger. These results make it clear that as opposed to natural Lys, the addition of reduced GSH disrupts the disulfide bond of Lys, resulting in a change in the conformation of Lys and the exposure of hydrophobic groups, enhancing the hydrophobic interaction between partially folded Lys [17,18].

### 3.2. Changes in the Molecular Structure of Lys

**Figure 7:**
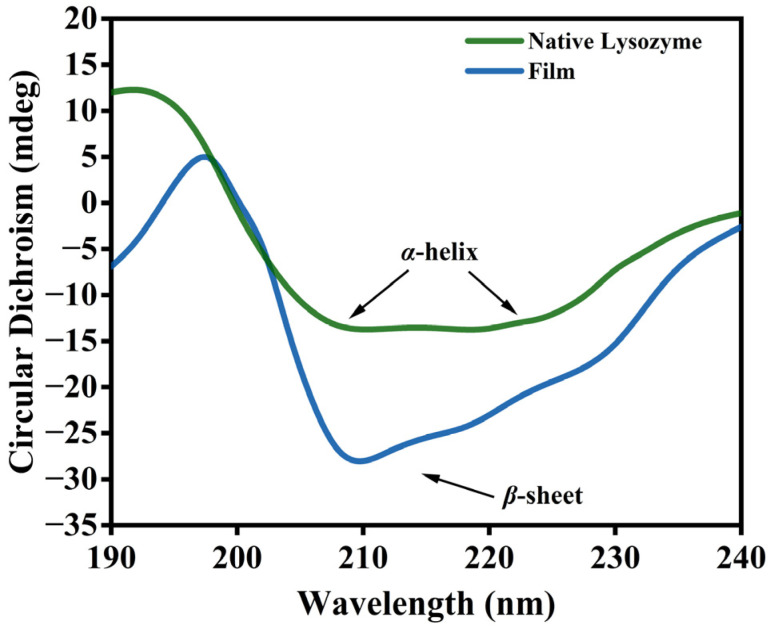
CD spectra of native Lys and the nanofilm.

CD is a fast, simple, and accurate method to study protein conformation in a diluted solution [19]. The spectrum displayed in Figure 7 shows that the α-helix structure peak of the natural Lys is at 220 nm and 208 nm. The α-helix characteristic peak of the Lys solution was replaced by the β-sheet characteristic negative peak (210 nm) following the reduction of GSH. These results demonstrated that the protein undergoes a transition from α-helix to β-sheets at the air/water interface during the formation of the lysozyme film [20,21].

**Figure 8:**
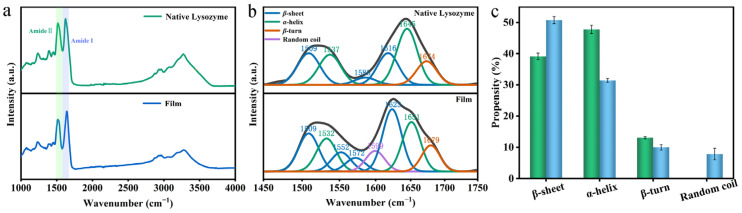
FTIR characteristic of the Lys nanofilm. (**a**) Comparison of the IR peaks between nanofilm and native Lys. (**b**) FTIR peaks of amide I and amide II of nanofilm, showing the deconvoluted β-sheet band. (**c**) Propensity analysis of the secondary structure of nanofilm and native Lys.

FTIR is a method used to inquire about changes in the intermolecular and intramolecular chemical bonds of Lys [22]. According to the infrared spectra of amide Ⅰ-Ⅱ (1500–1700 cm^−1^) in the protein film (Figure 8b), the structural characteristic peaks of native Lys were α-helix at 1537 cm^−1^, 1645 cm^−1^, β-sheet at 1509 cm^−1^, 1585 cm^−1^, 1616 cm^−1^, and β-turn at 1674 cm^−1^. In contrast, the protein film exhibited a wider characteristic peak of amide Ⅰ-Ⅱ, at 1532 cm^−1^ and 1651 cm^−1^ for a-helix, and in the range of 1509 cm^−1^, 1552 cm^−1^, 1572 cm^−1^, and 1623 cm^−1^ for β-sheet. At 1679 cm^−1^, the β-turn structure reached its highest point, and the typical peak of random coil structure was at 1599 cm^−1^. According to the outcome of the calculated secondary structure content of Lys, it was manifested that compared with the natural Lys, the content of β-sheet in the Lys films ascended to 50%, while that of α-helix descended to 31% as well as down to 10% for β-turn. The aforementioned results indicated that the secondary structure of the film shifted from α-helix to β-sheet. This observation was mainly due to the change in surface tension, which caused the protein molecules to cluster together [23], and the induction and drive of the intermolecular interaction; the β-sheet was more likely to accumulate at the interface to form a dense layer [24].

### 3.3. Lys Molecular Interaction

**Figure 9:**
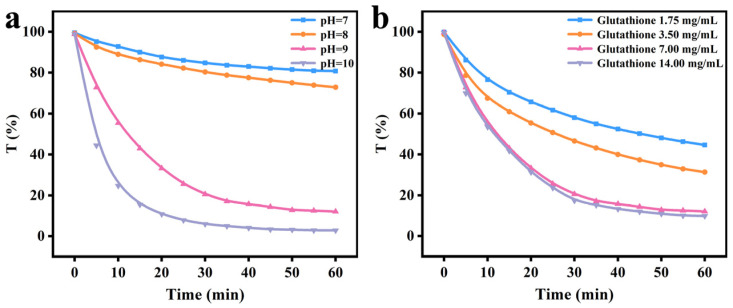
Spectrum of turbidity at 590 nm (transmittance %) as a function of the reaction time at four different pH values (**a**) and GSH concentrations (**b**).

As a certain amount of reduced GSH was added to the HEPES buffer solution of Lys, the GSH broke partial disulfide bonds and partially unfolded in a neutral and alkaline environment, which impaired the thermodynamic equilibrium of the system. Molecular aggregation of partially unfolded Lys is primarily responsible for hydrophobic interaction [25,26]. A 1 h turbidity test of unfolded Lys is illustrated in Figure 9a. At the point when pH was at 7 or 8, the level of ionization of Lys was low, and feeble aggregation occurred, thus diminishing its transmittance by approximately 20% after 1 h. As pH reached 9 or 10, the system produced visible aggregates, bringing a sharp decline in its transmittance. At a pH of 10, the Lys/GSH mixture presented a negative charge, the turbidity of the solution was the highest, and the permeability was almost zero. The closer to the isoelectric point of Lys (pI = 10.8), the more aggregates formed in the reaction system. Simultaneously, the growing GSH concentration gave rise to accelerated Lys foldability and lessened transmittance, signifying that the turbidity of the reaction system was more influenced by concentration (Figure 9b).

**Figure 10:**
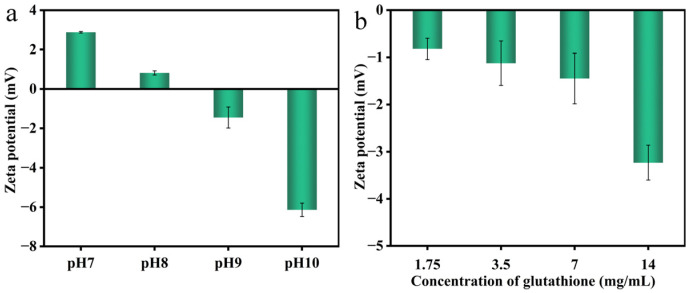
Effect of pH value (**a**) and GSH concentration (**b**) on the colloidal zeta potential in the solution incubated at 25 °C.

Zeta potential is a gauge of the surface charge of a protein molecule. Based on the results presented in Figure 10a, as pH was between 7 and 8, the electrostatic repulsion generated by the net positive charge on the particle surface resisted the aggregation of some colloidal particles. Whereas pH was equivalent to 9, the absolute value of the potential was at a low level, showing that the system clumped together, as the colloidal particles as a whole had a less negative charge and tended to attract each other. This was consistent with the outcomes revealed in Figure 10b, as GSH concentration improved in alkaline conditions (pH = 9), significantly elevating the protein molecule’s overall negative charge. This could be attributed to the rapid defolding rate of deregulation under the condition of sulfhydryl/disulfide bond exchange controlled by pH and GSH; the interaction between protein molecules was the strongest at this time, thereby gathering a great many nucleating particles [18].

**Figure 11:**
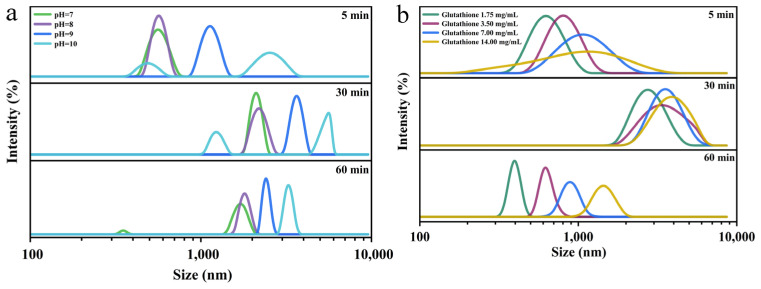
Effect of pH value (**a**) and GSH concentration (**b**) on particle size and its distribution in Lys-GSH buffer.

Figure 11a displays the size of nanoparticles at various pH values (pH = 7–10). The unfolded Lys monomers gathered quickly, and the particle size tended to increase as the pH value rose. However, the overall particle size was on an uptrend after 30 min, in contrast to the initial reaction, as the reaction time was prolonged even though the nanoparticle size followed a slight downtrend after 1 h reaction compared to 30 min. This could be related to the fact that the smaller particles adsorbed to the interface, while the larger ones formed precipitates. There was a single peak appearing after the reduction of Lys molecules by GSH at distinct concentrations (Figure 11b). Within 1 h of the reaction, the particle size also exhibited a trend of first increasing and then decreasing with time. The cumulative scattered light intensity tested by DLS revealed that the single peak shifted to the left with the passing of reaction time, and the particle size of the polymerization was distributed at 1000 nm, which may have been the particle size of the single molecule aggregates, mainly manifested as intramolecular association products. The addition of GSH enhanced the binding of hydrophobic groups and impaired the electrostatic repulsion, thus forming aggregates with large nanoparticles and settling under gravity [27,28]. This can be attributed to the fact that the polymer molecular chains would generate spatial curl, and the strength of intermolecular association was weakened, coupled with the shrinkage in corresponding particle size.

**Figure 12:**
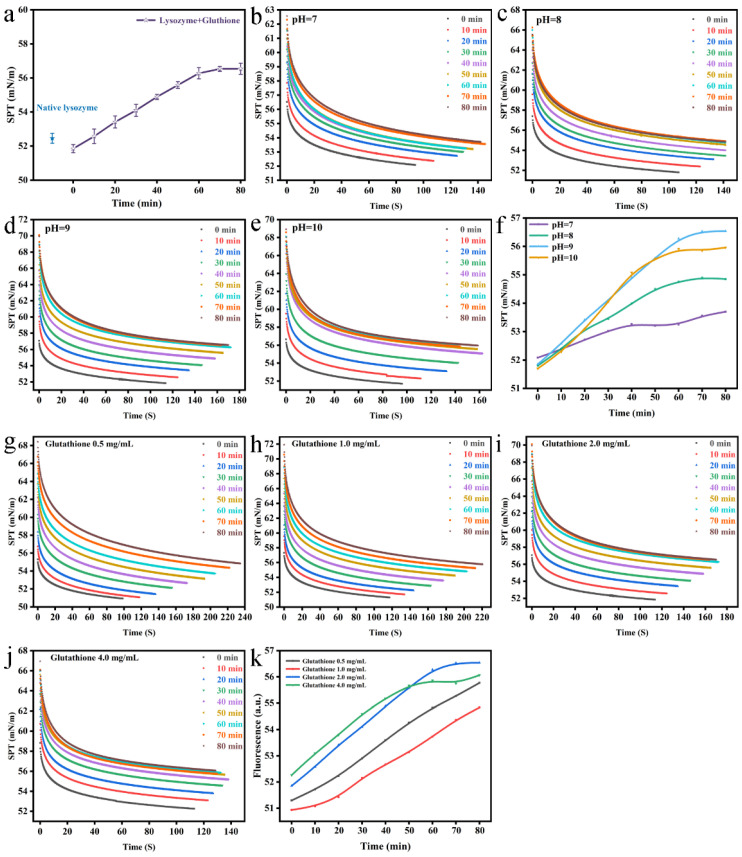
Change in surface tension of the Lys-GSH buffer as a function of reaction time (**a**). Change in surface tension of the Lys-GSH buffer as a function of reaction time at four different pH values (**b**–**f**) and GSH concentrations (**g**–**k**).

The changes in surface tension of folding Lys are presented in Figure 12. The earlier literature demonstrated that Brownian diffusion is the motive force in early interfacial tension diffusion [29,30]. Over time, the colloidal particles that had been adsorbed at the interface would prevent the particles in the continuous phase from adhering to the particle surface, consequently bringing down the surface tension. In this experiment, the hanging sheet method was used to test the surface tension of the mixed system of Lys and reduced GSH. The surface tension of the solution was 51.7 mN/m with the addition of reduced GSH, which was lower than that of the natural Lys (52.3 mN/m). Compared with the natural Lys, the surface tension of the solution after adding GSH notably declined, which may have been due to the aggregation of partially folded Lys molecules adsorbed to the surface interface [31]. In the process of aggregation, the aggregates on the surface were gradually consumed as reaction time progressed, presumably resulting in the moderate elevation of surface tension until achieving an equilibrium state (56.2 mN/m) (Figure 12a). The impacts of different pH values and reduced GSH concentrations on interfacial tension were investigated. As demonstrated in Figure 12f, as the pH rose, it was in a more alkaline condition such that as the Lys disintegration rate accelerated, the particles in the bulk phase adhered to the interface more rapidly and the interfacial tension of the Lys-reduced GSH mixture had an evident escalating tendency. The surface tension of unfolding lysozyme was enhanced with increasing glutathione concentration, with the other conditions unchanged (Figure 12k).

### 3.4. Characterization of Nanofilms

**Figure 13:**
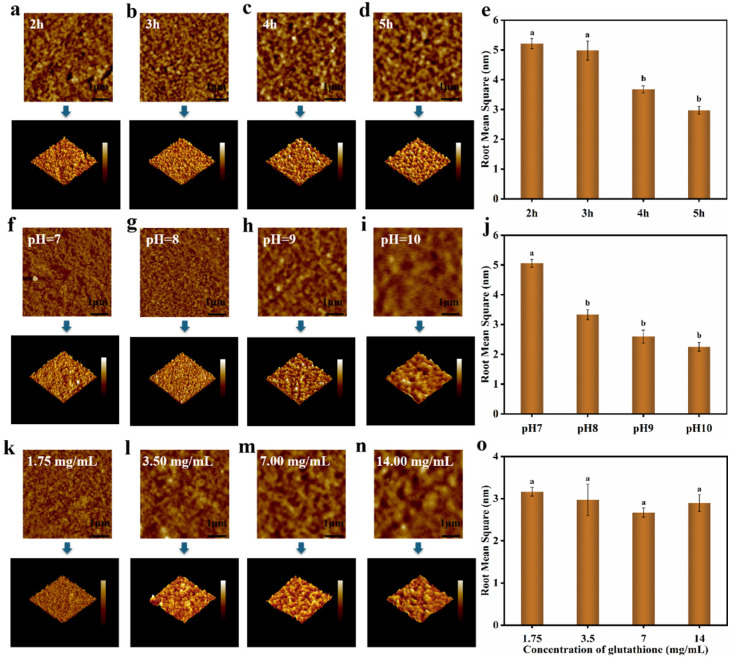
AFM measurements on the Lys/GSH nanofilm coated on glass wafer at different times in the GSH buffer. (**a**–**d**) AFM images of the nanofilm at 2 h, 3 h, 4 h, and 5 h. (**e**) The effect of time in the GSH buffer on the RMS of the nanofilm. The Lys/GSH nanofilm was obtained by incubating the mixture of Lys buffer (7 mg/mL in HEPES) and GSH buffer in equal volume 1:1 for different durations at 25 °C; AFM measurements on the Lys/GSH nanofilm coated on glass wafer at different pH values of the GSH buffer. (**f**–**i**) AFM images of the nanofilm at pH values of 7, 8, 9, and 10. (**j**) The effect of the pH of the GSH buffer on the RMS of the nanofilm. The Lys/GSH nanofilm was obtained by incubating the mixture of Lys buffer (7 mg/mL in HEPES) and GSH buffer (7 mg/mL in HEPES) in equal volume 1:1 for 3 h at 25 °C; AFM measurements on the Lys/GSH nanofilm coated on glass wafer at different concentrations of the GSH buffer. (**k**–**n**) AFM images of the nanofilm at the GSH concentrations of 1.75 mg/mL, 3.5 mg/mL, 7 mg/mL, and 14 mg/mL. (**o**) The effect of the GSH buffer concentration on the RMS of the nanofilm. The Lys/GSH nanofilm was obtained by incubating the mixture of Lys buffer (7 mg/mL in HEPES) and GSH buffer (7 mg/mL in HEPES) in equal volume 1:1 for 3 h at 25 °C.

As the growth rate of surface tension slowed down, a complete film tended to form at the interface so as to achieve a new thermodynamic equilibrium [32]. The particles already clinging to the interface were capable of protecting the particles in the continuous phase from sustaining the adsorption to the interface. Lys nanofilms were gathered by the aggregation and accumulation of single-layer particles, for which reason a smooth surface morphology had the potential to be accomplished through the assembly process of rapid nucleation followed by gradual aggregation and fusion [33,34]. The influence of different conditions on the morphology of nanofilms was investigated by AFM. Firstly, we explored the effect of inoculation time on the morphology and roughness of the film, as shown in Figure 13e. The Lys film could be promptly assembled at the interface within 2 h. The morphology of the Lys film was packed more tightly with the passage of time, and its surface became smoother, which might have been conducive to shaping into a protein coating formed by the compact accumulation of oligomers. In parallel, the morphology and roughness of the Lys film were examined in relation to the pH value and GSH concentration, respectively. It can be noted that as the pH of the reaction system rose (Figure 13f–j), the packing became denser as larger oligomers were formed; thus, the roughness of the films decreased in the same period of incubation time. The higher the concentration of reduced GSH (Figure 13k–n), the faster the particles in the bulk phase aggregated over reaction time, together with the fast and tight assembly carried out at the interface [35,36].

## 4. Conclusions

In summary, it is possible that sulfhydryl/disulfide bond exchange causes lysozyme to unfold when GSH breaks the disulfide bond in the molecule, which leads to changes in the conformation of the lysozyme molecule involved in the increased β-sheet structure and random coil content. Further structural changes enabled unfolded lysozyme to form interfacial films at the air/water interface by altering intermolecular interactions and assembly modes. GSH concentrations or pH values could be adjusted to regulate the lysozyme molecular folding process and assembly of interface membranes as well as the microstructure of the interface membrane. This study not only gives insight into the intrinsic mechanism of GSH-induced lysozyme interface assembly, but it also provides direction and guidance for future research grounding on the interfacial assembly behavior of unfolded lysozyme. How to enhance the adhesion strength between Lys nanofilms and substrates and simplify their preparation process are still urgent topics for future exploration.

## Data Availability

Not applicable.
